# Some Points on Prudence and Jurisprudence

**Published:** 1906-07

**Authors:** W. A. L. Knowles

**Affiliations:** San Francisco, Cal.


### SOME POINTS ON PRUDENCE AND JURISPRUDENCE.

By W. A. L. Knowles, M. D., D. D. S., San Francisco, Cal.

It is well to obtain and preserve models of all cases of orthodontia previous to commencing the operation, at intervals during the progress and on its completion. These relate their own stories, often in direct contradiction to the memory of the other interested parties. Disputes sometimes arise, but the models are mute and unimpeachable evidence.

If you are so unfortunate as to break off a tooth in an attempted extraction, leaving a portion of the root or roots in the alveolar process, retain the broken portion of the tooth and place it in an empty vial (Gold foil bottles answer admirably) with the name of the patient and date of operation on a small slip of paper also enclosed.

This is also unimpeachable evidence and later, if the other portion is recovered, the portions may be assembled to assure one of the recovery of all fragments.

Make copious notes upon your record book of all peculiar conditions attending an operation such as dead nerves, number, condition and general direction of canals, absence of canals, exposed nerves, location of such exposure and material used for capping or covering.

Note pathological conditions, if any, of the gingiva or the peridental membrane.

Note anything abnormal in the vicinity of the operation performed.

Note loosened teeth, pyorrhoea pockets, receded gums, cervical caries, soft spots, chalky islands, stains and effects of tartar deposits.

Note the impossibility of retaining a clamp upon the tooth and also the failure to retain the rubber dam in position.

Make a note of any specific instructions delivered to the patient at the time of the performance of the operation.

In long operations, make a note of the length of time consumed, recording both the hour of commencement and the time of its completion.

In case of the accidental loss of a portion of a drill or broach in a tooth and finding its removal impossible, make copious notes of the facts.

When a peculiar history of a case is related to you by the patient, perhaps relating to the treatment or method used by some other dentist or physician, make notes of such statements, with names and dates when possible to obtain them.

Retain copies of all important letters and papers sent out to patients, parents or guardians of patients or professional associates.

When making an estimate upon the contemplated cost of any operation, if such request is made and acceded to, be very careful to make a note of such estimate in a book kept for that purpose.

Do not adversely criticize the work of your professional associates in the presence of the patient, unless it exhibit rank malpractice or indicate the grossest igorance or carelessness.

Be on your guard all the time when using any corrosive substance, about the patient,, that none may, by any mischance, come in contact with the body or clothing.

Note the presence of chronic abscesses or old fistulae.

Note canal perforation if so unfortunate as to produce it.

Note suspected antral trouble.

Note unerupted teeth when long past the time for their eruption.

Note retained deciduous teeth.

Note time of insertion of all temporary fillings and the fact that the patient was so notified.

An operation is occasionally modified to suit the patient, even against the best judgment of the operator. In such a case, make copious notes and also be sure to note the fact that such operation was so modified at the special request of the patient, who assumed all liability therefor, having been informed as to the possible consequences.

Keep your record books accurate, marking the exact location of all operations, the materials used and the date when performed.

Accurately note the condition of the nerve and other interesting points on all portions of teeth covered by shell crowns which conceal the parts from view.

Before pouring from any bottle containing any preparation which you intend using, be careful, in every instance, to carefully examine the label.

Buy the best drugs of the most reliable manufacturers, especially those used in anaesthetic operations.

Be careful not to use wood alcohol, which is poisonous, when grain alcohol is required.

Take the best care of forceps, scalers, clamps and hypodermic syringes and needles.

Do not use the rubber dam more than once, even upon the same patient.

Be careful of ligatures and do not use them on patients a second time.

Be exceedingly careful in the use of arsenious acid or preparations containing arsenic, and always hermetically seal the application in the cavity, always applying the rubber dam when possible.

Be careful of ligatures about the teeth when using carbolic, acetic, lactic or trichloracetic acids or other corrosive liquids as they often become soaked and lead the liquids to the live tissues where considerable damage may be done.

Be careful in passing any sharp instrument over the patient's head to guard the point or edge, being especially watchful of the eyes.

Assume no unnecessary risks.

Be very careful to thoroughly sterilize the mouth pieces of the saliva ejector and all burs and instruments, bearing in mind the fact that the saliva carries infection. The most serious danger is the liability to innoculate with the virus of tuberculosis or syphilis. So called fever sores or cold sores are sometimes carried from mouth to mouth by means of the imperfectly sterilized mouthpiece of the saliva ejector. It is almost wonderful that the rubber dam did not cause more serious results than have been reported when we consided that, in the early days of the introduction of the rubber dam, it was used on patient after patient until it became riddled with holes, appearing as if it had been used as a shot-gun target and when we also consider the fact that the dental journals of those days published items from practitioners furnishing information as to how those holes could be filled up and the rubber used again.

Do not vouch for new remedies until they have been thoroughly tried and have been proven to be efficacious and safe.

Be cautious and conservative.

Be not too hasty in diagnosis.

Use nothing for the reason that it is cheap. Remember that good instruments command good prices.

Do not indulge in eating onions or garlic on the day you operate or the day preceding.

It may be more easy to refrain from using nostrums than to explain to the plaintiff's attorneys in a malpractice suit or to the coroner, that you did not know the ingredients of the preparation used.

Do not leave the cleaning and sterilizing of instruments to assistants. Do it yourself and you know how it is done. Forceps or clamps borrowed from dentists who depend on assistants to care for them, will sometimes demonstrate the wisdom of this advice.

Keep your drinking glass clean.

Always disinfect canals of the teeth before passing instruments into them.

As far as possible, hold only business conversation over the telephone during business hours.

Instruct your patients to leave no valuables in the unattended reception room during their temporary absence in the operating room.

Be sure to have safety fuses between your office apparatus and the electric current.

Be careful to have ready for immediate use ant-acids, glycerine, vaseline and disinfectants.

During anaesthetic operations always have nitrite of amyl pearls and a tongue forceps at hand.

Have a fan handy and also a pan in case of sudden emesis.

Ammonia must be within ready reach and a bottle of whiskey or brandy should be a part of the necessary paraphernalia of every operating room.

Never give an anaesthetic to a female except in the presence of a third party, and it is always advisable to have one attend to the anaesthetic while a second one performs the operation...

Be careful not to be too optimistic in promising success in operations in which the outcome is not assured.

In orthodontia cases, when moving the teeth, be careful to carefully calculate the amount of resistance required from those teeth used as points for anchorage.

Be careful of alcohol lamps and do not allow the unguarded flame to approach inflammable materials worn by children or lady patients.

Use pointed fissure burs with extreme caution in forward cutting.

In using disks, cut away from the lips, reversing the engine when using it upon the left side of the mouth and also interpose the finger or thumb between the disk and the soft tissues so that if the disk should catch and jump, it would not injure the patient.

Guard against sudden movements which might startle patients and be especially careful with patients having organic disease of the heart.

Be careful not to allow any irritating medicaments to run backward down the throat. Sometimes these produce temporary sore throat by affecting muscles having a point of attachment near the portion irritated.

In obtaining plaster impressions of the mouth, cause the patient to depress the chin and move the head forward so that no fragment of plaster may escape down the throat.

In extracting a number of roots it is a wise plan to mark their positions upon a diagram previous to operating and to check them off as fast as the extraction proceeds. This enables one to know when the operation is completed and also prevents the necessity of searching for buried roots amid the blood which floods the mouth and masks the location of those last remaining in situ.

